# Immunogenicity Studies of Bivalent Inactivated Virions of EV71/CVA16 Formulated with Submicron Emulsion Systems

**DOI:** 10.1155/2014/670506

**Published:** 2014-06-11

**Authors:** Chih-Wei Lin, Chia-Chyi Liu, Tsung-Chun Lu, Shih-Jen Liu, Yen-Hung Chow, Pele Chong, Ming-Hsi Huang

**Affiliations:** ^1^National Institute of Infectious Diseases and Vaccinology, National Health Research Institutes, Zhunan Town, Miaoli County 35053, Taiwan; ^2^Graduate Institute of Immunology, China Medical University, Taichung 40402, Taiwan; ^3^Biotechnology Center, National Chung Hsing University, Taichung 40227, Taiwan

## Abstract

We assessed two strategies for preparing candidate vaccines against hand, foot, and mouth disease (HFMD) caused mainly by infections of enterovirus (EV) 71 and coxsackievirus (CV) A16. We firstly design and optimize the potency of adjuvant combinations of emulsion-based delivery systems, using EV71 candidate vaccine as a model. We then perform immunogenicity studies in mice of EV71/CVA16 antigen combinations formulated with PELC/CpG. A single dose of inactivated EV71 virion (0.2 **μ**g) emulsified in submicron particles was found (i) to induce potent antigen-specific neutralizing antibody responses and (ii) consistently to elicit broad antibody responses against EV71 neutralization epitopes. A single dose immunogenicity study of bivalent activated EV71/CVA16 virion formulated with either Alum or PELC/CpG adjuvant showed that CVA16 antigen failed to elicit CVA16 neutralizing antibody responses and did not affect EV71-specific neutralizing antibody responses. A boosting dose of emulsified EV71/CVA16 bivalent vaccine candidate was found to be necessary to achieve high seroconversion of CVA16-specific neutralizing antibody responses. The current results are important for the design and development of prophylactic vaccines against HFMD and other emerging infectious diseases.

## 1. Introduction

Human enterovirus (EV) 71 and coxsackievirus (CV) A16 are the two most common etiologic agents of hand, foot, and mouth disease (HFMD) in infants and young children [1–8]. They have especially emerged as important neurotropic viruses in the Asia-Pacific region, where they threaten to be coined the new polio [1–4]. However, no specific antiviral therapy or vaccine is currently available for the nonpolio enteroviruses; general cleanliness and school closures are probably effective in reducing and controlling the spread of these viruses [1]. Presently, five institutes (National Health Research Institutes, Taiwan; Sinovac Biotech, China; Beijing Vigoo Biological, China; Chinese Academy of Medical Sciences, China; and Inviragen, Singapore) have produced chemically inactivated EV71 virion candidate vaccines using different EV71 vaccine strains and different manufacturing technologies and tested them in human clinical trials [4–8]. A notable advance in phase III clinical trial in China demonstrated that the inactivated EV71 vaccine elicited EV71-specific immune responses and provided protection against EV71-associated diseases including HFMD, herpangina, and neurological complications; among more than 30,000 healthy children received two doses of placebo or EV71 vaccine candidate [6–8]; however, the scientists found no evidence that the EV71 vaccine provided protection against CVA16. Recently, the findings that CVA16-specific neutralizing antibodies generated from mice vaccinated with inactivated CVA16 virions could not cross-neutralize EV71 virus were reported in the literature [9, 10]. Taking these results together, a bivalent vaccine containing antigens from both EV71 and CVA16 viruses is urgently needed to eliminate HFMD. In addition, the use of a vaccine adjuvant capable of inducing potent and broadened immune responses is another potential strategy to overcome the chemical and immunological incompatibility interference in the vaccines combination.

Among the vaccine adjuvants evaluated in human trials, oil-in-water (O/W) emulsion systems are found to have the most significant potential in massive/emergency vaccination [11]. O/W emulsions possess better efficiency than the conventional aluminum-based mineral adjuvant (generally termed Alum) due to the induction of cytokines, cytokine receptors, and genes involved in leukocyte migration and antigen presentation at the site of injection [11, 12]. Previously, we designed and optimized a submicron multiphase emulsion comprised of the bioresorbable polymer, Span 85, and squalene forming a ready-to-use adjuvant, called PELC, which has an interesting physical feature water-in-oil-in-water (W/O/W) [13, 14]. Moreover, recombinant H5N1 trimeric HA influenza protein coimmunized with PELC plus immunostimulatory CpG molecules induces robust immunity [15, 16]. We also substantiated that the emulsified submicron particles could overcome the immunologic interference between the antigens and enhance the antigen-specific immune responses, suggesting a 3-in-1 vaccine against enterovirus, influenza virus, and/or Japanese encephalitis virus via microencapsulation technology [17].

In this study, we propose two approaches to develop an effective bivalent EV71/CVA16 vaccine formulation against HFMD. The bivalent vaccine candidate would be formulated with PELC emulsion system with and/or without CpG and tested in mice for induction of humoral responses following a single-dose or a prime/boost vaccination schedule. The results were compared with those obtained either with conventional Alum suspensions or without adjuvant.

## 2. Material and Methods

### 2.1. Vaccine Preparation

The vaccine compositions comprised a EV71 formalin-inactivated whole virus derived from the clinical isolate, E59 strain (genotype B4, kindly given from the Taiwan CDC), and an inactivated CVA16 vaccine derived from a type A16 coxsackie strain (N5079 clinical isolate, obtained from National Cheng-Kung University, Taiwan). Both EV71/CVA16 viruses were propagated in African green monkey kidney (Vero) cells in the cell-based serum-free perfusion bioreactor process. The EV71/CVA16 vaccine bulks were obtained after sterile filtration using a 0.22 *μ*m-filter, subjected to SDS-PAGE and western blot analyses, and stored at 4°C. The total protein concentration of the vaccine bulk was determined by the bicinchoninic acid (BCA) method protein assay. Viral titers were determined using the median endpoint of the TCID_50_ (50% tissue culture infective dose) by counting the cytopathic effects on infected Vero cells. Production details for the EV71/CVA16 vaccine candidates are reported elsewhere [5, 9, 18].

### 2.2. Adjuvant Preparation

Murine CpG oligodeoxynucleotide was synthesized by Invitrogen Taiwan Ltd. and given as a 10 *μ*g per dose dissolved in the sterile water. The CpG sequence used was 5′-TCC ATG ACG TTC CTG ACG TT-3′ with all phosphorothioate backbones. Alum adjuvant (aluminum phosphate suspension) was kindly provided from Taiwan CDC and given as a 300 *μ*g per dose in the acidic media (pH = 6). PELC is a squalene W/O/W nanoemulsion stabilized by Span 85 (sorbitan trioleate, Sigma-Aldrich, Steinheim, Germany) and bioresorbable polymer, poly(ethylene glycol)-block-poly(lactide-co-*ε*-caprolactone) (PEG-b-PLACL) [13, 14]. Briefly, aqueous solution comprising 120 mg PEG-b-PLACL and 0.8 mL phosphate buffer saline (PBS) and oily solution consisting of 0.935 mL squalene (Sigma-Aldrich, Steinheim, Germany) and 0.165 mL Span 85 were emulsified using Polytron PT 3100 homogenizer (Kinematica AG, Switzerland) under 6,000 rpm for 5 min. PEG-b-PLACL was replaced by the same amount of Tween 80 in the aqueous solution so as to yield PELC(T) emulsion. In parallel, the excipients, PEG-b-PLACL and Span 85, used in the aqueous solution and oily solution of PELC were substituted for Tween 80 and *α*-tocopherol so as to render PELC(A) emulsion, respectively. The emulsified formulations were stored at 4°C until use. Emulsion-formulated vaccines were investigated by redispersing 200 *μ*L of stock emulsion into 1800 *μ*L of EV71/CVA16 vaccine bulks and were mixed with a test-tube rotator (Labinco LD-79, Netherlands) under 5 rpm at least 1 hr before injection. The emulsion adjuvant was given at 20 *μ*L per dose. The size distribution of the formulations was determined using laser light scattering technique (Brookhaven 90 plus particle size analyzer, Brookhaven Instruments Limited).

### 2.3. Peptide Synthesis

VP1_211–225_ (peptide sequences: FGEHKQEKDLEYGAC) and VP2_136–150_ (peptide sequences: AGGTGTEDSHPPYKQ) were synthesized in-house by solid phase method using an automated multichannel parallel peptide synthesizer (model Prelude from Protein Technologies, Inc.), employing the fluorenylmethoxycarbonyl (Fmoc) group for *α*-amino group protection. Mass spectrometry data obtained from Agilent 1100 Series LC/MSD high performance ion trap mass spectrometers to ensure the target peptide is obtained. The purity was >90% when used.

### 2.4. Ethics Statement and Vaccination

All experiments were conducted in accordance with the guidelines of laboratory animal center of NHRI. The animal use protocols have been reviewed and approved by the NHRI Institutional Animal Care and Use Committee (NHRI-IACUC-099073-A). Five-week-old female BALB/c mice were obtained from the National Laboratory Animal Breeding and Research Center (Taipei, Taiwan) and acclimatized for at least one week at the NHRI animal facility prior to use. Two hundred microliter of candidate combination vaccine was intramuscularly (i.m.) administered to mice in either one-dose or two-dose schedule. Serum samples were collected from vaccinated mice via the submandibular veins and the antibody titers were determined by enzyme-linked immunosorbent assay (ELISA) and viral neutralizing (VN) assays.

### 2.5. ELISA Immunoassay

The presence of specific antibodies in the sera was determined by ELISA. In brief, 100 *μ*L of dilute inactivated virus (EV71 or CVA16; 2 *μ*g/mL) or epitope peptide (VP1_211–225_ or VP2_136–150_; 5 *μ*g/mL) was coated in 96-well microtiter plates with 0.05 M carbonate buffer (pH 9.6, Sigma, St. Louis, MO, USA) by overnight incubation at room temperature. Coated plates were washed once with PBS containing 0.05% Tween 20 (Sigma, St. Louis, MO, USA) and then blocked with 1% bovine serum albumin (BSA, Sigma, St. Louis, MO, USA) in PBS at room temperature for 2 h. Diluted sera (starting dilution 1 : 1000, serial two-fold serum dilutions) from vaccinated animals were applied to wells at room temperature for 2 h. Followed by HRP-conjugated goat anti-mouse IgG (ICN Cappel, Aurora, Ohio, USA, 1 : 5,000), the assay was developed with tetramethylbenzidine chromogen substrate (NeA-blue clinical science products Inc., MA, USA) for 20 min at room temperature (avoid light). Plates were read at 450 nm using an ELISA plate reader (Molecular Devices, Sunnyvale, CA, USA). The titers were determined from the reciprocal of the final dilution that gave an optical of two-fold absorbance of preimmune sera. For calculation purposes, an undetectable level was scored as a titer equal to 500.

### 2.6. VN Assay

To provide a more functional measure of vaccine-induced immunity, 200 TCID_50_ per well of virus were incubated with two-fold-diluted mice sera at a starting dilution of 1 : 20. Mixtures of virus and serum were transferred to monolayer of cells (EV71: Vero cells; CVA16: rhabdomyosarcoma cells) and incubated at 37°C and 5% CO_2_ for 7 days. The neutralizing titer was defined as the reciprocal of the highest serum dilution at which the infectivity of 200-TCID_50_ virus was completely neutralized in 50% of the wells. Infectivity was identified by counting cytopathic effects (CPF) in infected cells and the titer was calculated using the Reed-Muench method. For calculation purposes, an undetectable level was scored as a titer equal to 10. The seroconversion rate was calculated either from the proportion of mice achieving a postvaccination titer four-fold increase as compared to day 0 or the proportion of mice with a postvaccination titer ≥1 : 40 as compared to a prevaccination VN antibody titer <1 : 10.

### 2.7. Statistical Analysis

The statistical analysis was conducted using GraphPad Prism version 5.02 (GraphPad Software, Inc.). Comparison of the antibody titers between groups was calculated by use of an ANOVA model followed by a Bonferroni posttest on log⁡_10_-transformed values.

## 3. Results

### 3.1. Formulating Inactivated EV71 with Emulsified Submicron PELC Particles

In order to enlarge the emulsion adjuvant system for the HFMD vaccine development, we prepared two additional emulsions on the basis of the composition of PELC (Table 1). These emulsion systems were denominated to be PELC(T) and PELC(A) when PEG-b-PLACL and Span 85 were replaced with Tween 80 and *α*-tocopherol, respectively (see Section 2.2). Tween 80 and Span 85 were selected as the emulsification agents because they were currently used in licensed human vaccines. The *α*-tocopherol was used as an excipient and immunostimulatory agent against antigen-presenting cells of the innate immune system.

PELC, PELC(T), and PELC(A) were prepared using homogenization process described in Section 2; each white and isotropic emulsion was rendered. The composition and particle size of the resulting emulsion systems were characterized and analyzed; the results are summarized in Table 1. Squalene-based emulsions were usually uniform with submicron in size (the particle size distribution between 300 and 400 nm) no matter what emulsifier/excipient was used in the formulations. The three emulsion systems were then investigated for their ability to enhance antigen-specific and cross-neutralizing immune responses in mice.

**Figure 1:**
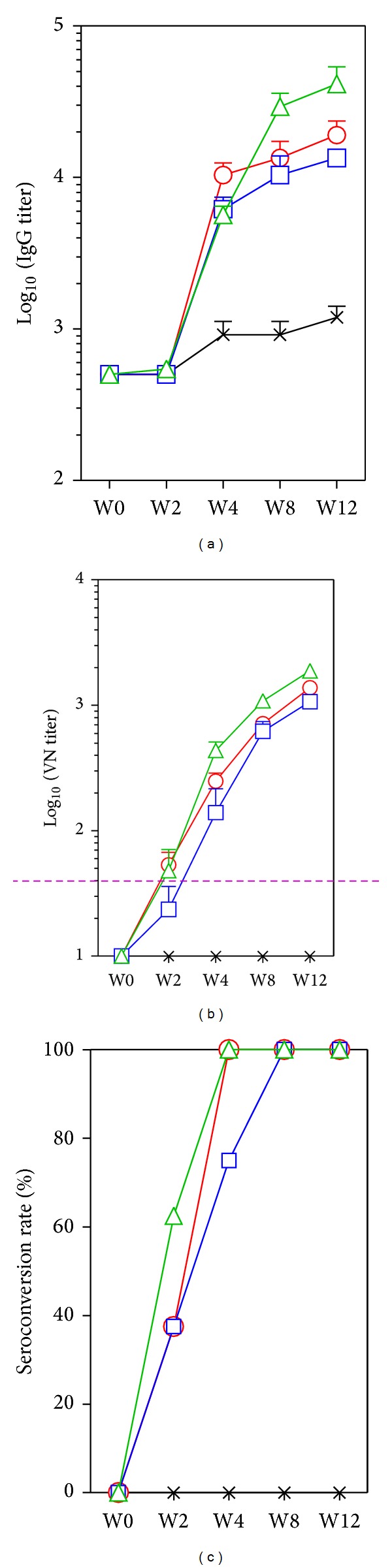
EV71 antigen-specific (a) IgG, (b) VN antibody responses, and (c) seroconversion rate in BALB/c mice vaccinated with a single dose of EV71 inactivated virus vaccine. Mice (*N* = 8) were injected i.m. with 0.2 *μ*g of inactivated EV71 virus alone or formulated with different emulsions: (-x-) no adjuvant, (-o-) PELC, (-□-) PELC(T), and (-∆-) PELC(A). Serum samples were collected from the vaccinated mice and the antibodies were measured by ELISA and VN. The data were presented as GMTs with standard errors of eight mice per group. The dotted horizontal line represents a VN titer of 40. The seroconversion rate is the percentage of mice achieving a postvaccination titer ≥40.

From our previous single-dose studies, 0.2 *μ*g of inactivated EV71 virion was found to be the minimum antigen that formulated with Alum could induce EV71 viral neutralizing antibody response [4, 19]. To evaluate whether EV71-specific antibodies could be elicited by a single-dose vaccination, BALB/c mice were injected with 0.2 *μ*g of inactivated EV71 virion either alone or formulated with individual PELC emulsion systems. The elicited antigen-specific antibodies are shown in Figure 1. Following the injection, sera from mice vaccinated with inactivated EV71 virion alone elicited EV71-specific IgG antibody geometric mean titers (GMT) less than 500 at week 2 (Figure 1(a)). Afterwards, it increased slowly to 900 at week 4 and week 8, and then to 1,200 at week 12. When the same amount of inactivated EV71 virion was coadministered with PELC, PELC(T), or PELC(A) emulsions, the induced anti-EV71 IgG titers were much stronger and higher than those induced by inactivated virus alone (*P* < 0.001 after 2 weeks postvaccination). There were no statistically significant differences in IgG titers between the three emulsion-formulated groups.

VN assays were also performed to provide a functional measure of vaccine-induced immunity.  Figure 1(b) shows VN antibody responses elicited in BALB/c mice vaccinated with inactivated EV71 virus, formulated with or without adjuvant. Overall, sera from mice vaccinated with 0.2 *μ*g of inactivated virion alone were found to have EV71-specific VN titer less than 20 after a single vaccination. This finding implied that a single-dose nonadjuvant inactivated EV71 vaccine at this dosage probably could not provide serological protection against the homologous virus challenge. In contrast, the same amount of inactivated virus was coadministered with PELC emulsion systems; mouse anti-EV71 neutralization titers were found to be significantly higher than those obtained with nonadjuvant inactivated virus (Figure 1(b)). The neutralizing antibody levels were induced progressively and probably had not reached the highest level until week 12. Considering the seroconversion rate (mice achieving a postvaccination VN titer higher than 40), only the mice received PELC or PELC(A)-emulsified candidate vaccines could reach 100% before 4 weeks (Figure 1(c)). Although PELC, PELC(T), or PELC(A)-emulsified EV71 candidate vaccines elicited potency immune responses against EV71, sera from mice vaccinated with these vaccines still failed to cross-neutralize CVA16. Since PELC, PELC(T), or PELC(A)-emulsified candidate vaccines have similar potency to correlate the results from previous studies, PELC emulsion system is selected for the following studies.

### 3.2. PLEC-Emulsified EV71 Candidate Vaccine Elicited Strong Neutralizing Antibody Responses against EV71 but Failed to Broaden Neutralizing Antibody Response against CVA16

**Figure 2:**
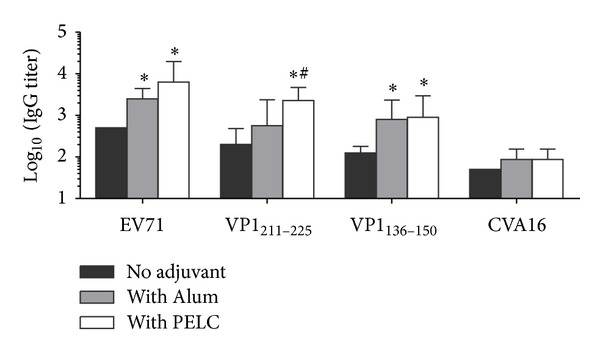
Antigen-specific IgG antibody responses in BALB/c mice vaccinated with a single dose of EV71 inactivated virus formulated with different adjuvant. BALB/c mice (*N* = 6) were i.m. vaccinated with 0.2 *μ*g of inactivated EV71 virus alone or formulated with Alum or PELC emulsified particles. At week 8, serum samples were collected from blood and the antibodies were measured by ELISA; the reactivity of neutralization epitope VP1_211–225_ or VP2_136–150_ was determined by peptide ELISA. The data were presented as GMTs with standard errors of six mice per group. _ _*P < 0.05: comparison with the group without adjuvant. ^#^ *P* < 0.05: comparison with the group of Alum adjuvant.

Our previous experiment [18] showed that two doses of 0.5 *μ*g of EV71 vaccine formulated with Alum could not elicit strong neutralizing antibody responses with GMT less than 200. To test whether PLEC-emulsified EV71 candidate vaccine had elicited broader neutralizing antibody responses against neutralization epitopes, antisera from mice vaccinated EV71 vaccine alone or formulated with wither Alum or PELC were tested against two known virus neutralization epitopes: VP1_211–225_ and VP2_136–150_ [19]. According to the literature, there was 60% and 90% homology between EV71 and CVA16 at the peptide residues VP1_211–225_ and VP2_136–150_, respectively [19]. As shown in Figure 2, both EV71 inactivated virion alone and Alum-formulated induced antibody responses with significant low IgG titer against VP1_211–225_ peptide compared those obtained with PELC-emulsified group (*P* < 0.05). However, no significant difference was observed between Alum- and PELC-formulated vaccines in the VP2_136–150_ peptide-specific antibody responses (Figure 2). The results so far suggest that PELC-emulsified EV71 vaccine elicits stronger and broadens antibody responses against EV71 neutralization epitopes than those formulated with Alum. Although there was >90% homology between EV71 and CVA16 at VP2_136–150_ peptide sequence, EV71-specific antibodies reacted poorly with CVA16 and failed to neutralize CVA16 at 1/20 dilution.

### 3.3. PELC/CpG Combination Adjuvant

**Figure 3:**
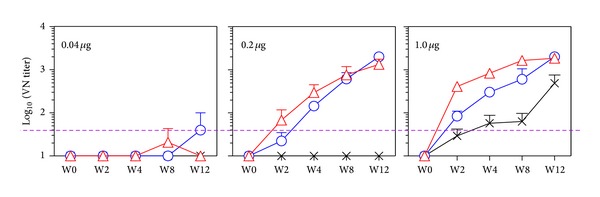
EV71-specific VN antibody responses in mice vaccinated with single dose of inactivated EV71 virus formulated with different adjuvant. BALB/c mice (*N* = 6) were vaccinated i.m. once with the candidate vaccine formulations: (-x-) no adjuvant, (-o-) PELC, and (-∆-) PELC/CpG. Blood samples were collected from vaccinated mice at different weeks and the antibody titers were determined by VN assays. Data are presented as mean titers with standard errors of six mice per group; the dotted horizontal line represents a VN titer of 40.

Certainly PELC-emulsified EV71 candidate vaccine easily and quickly elicited 100% of seroconversion against a homologous virus strain and enhanced antibody titer against the immunodominant neutralization epitopes of EV71. In our previous studies, the potency of PELC could be significantly increased by combining CpG, a well-known adjuvant. As shown in Figure 3, the EV71-neutralizing antibody responses in mouse group vaccinated single dose with either 0.04 *μ*g or 0.2 *μ*g of nonadjuvant inactivated EV71 virion were found to be undetectable (<1/20). When 1 *μ*g of nonadjuvant EV71 candidate vaccine was used as the single vaccination dose, mouse VN titer was detected at week 2 (GMT = 32). Afterwards, the GMT was found to be 49, 54, and 490 at weeks 4, 8, and 12, respectively. Interestingly, EV71 candidate vaccine formulated either with PELC or PELC/CpG, VN GMTs were significantly enhanced in both 0.2 *μ*g and 1.0 *μ*g groups as shown in Figure 3 (*P* < 0.001). In contrast, a single dose of 0.04 *μ*g of EV71 candidate vaccine formulated either with PELC or PELC/CpG, the anti-EV71 VN titer, was not detected (<1/20) during the whole 12 weeks study. This finding implies that a single-dose PELC/CpG-emulsified 0.04 *μ*g of EV71 candidate vaccine probably could not provide serological protection against the homologous virus. It is worth mentioning that when the vaccination dose was increased from 0.2 *μ*g to 1.0 *μ*g, the VN titer was observed as early as week 2 and reached the plateau at week 8. Also, the neutralizing antibody responses induced by PELC/CpG-formulated EV71 vaccine were higher than those induced by PELC-formulated vaccine in the early stage of the postvaccination (Figure 3). Taking the results together, the synergistic adjuvant effect was observed when PELC combined with CpG.

### 3.4. EV71/CVA16 Bivalent Vaccine

**Figure 4:**
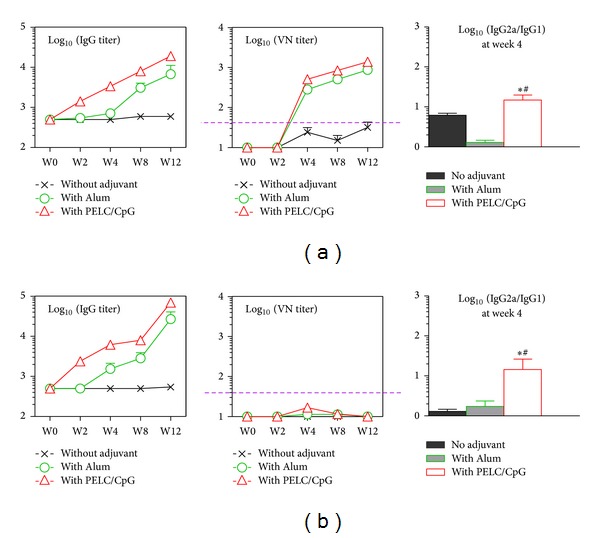
(a) EV71-specific and (b) CVA16-specific antibody responses in mice vaccinated with inactivated EV71/CVA16 combination vaccine. BALB/c mice (*N* = 6) were vaccinated i.m. once with different candidate formulations containing 0.2 *μ*g of EV71 and 0.2 *μ*g of CVA16. Serum samples were collected from vaccinated mice and the antibody titers were determined by immunoassays. Data are presented as geometric mean titers with standard errors of six mice per group. The dotted horizontal line represents a VN titer of 40. _ _*P < 0.05: comparison with the groups without adjuvant at the same time point. ^#^ *P* < 0.05: comparison with the group of Alum adjuvant at the same time point.

We have previously performed the immunogenicity study of an inactivated CVA16 whole-virion vaccine formulated with Alum in mice [9]. To broaden the immune responses against HFMD, we performed mouse immunogenicity studies to examine the efficacy of a bivalent EV71/CVA16 candidate vaccine by incorporating formalin-inactivated CVA16 virion into EV71 vaccine with and/or without adjuvant. As expected, sera from mice vaccinated with single dose of bivalent candidate vaccine contained 0.2 *μ*g of each virion alone (nonadjuvant); both EV71 and CVA16 virions elicited little or no functional antibody responses (Figure 4). Both anti-EV71 and anti-CVA16 specific IgG titers were found to be significantly increased after 8 weeks postvaccination with single dose of Alum formulated bivalent vaccine. In addition, PELC/CpG-formulated bivalent vaccine was found to elicit the highest IgG antibody titers against both EV71 and CVA16 virus. This single low-dose vaccination also reveals the advantage of vaccination with PELC/CpG-formulated bivalent vaccines that allows the host to produce a bias IgG2a/IgG1 ratio (*P* < 0.001). The bivalent EV71/CVA16 vaccine formulated with either Alum or PELC/CpG had induced excellent VN titers against EV71 (GMT higher than 200 after 2 weeks postvaccination), but to our surprise failed to elicit neutralizing antibody responses against CVA16 (Figure 4(b)). This result is consistent with our previous study that CVA16 is less immunogenic than EV71 [9].

**Figure 5:**
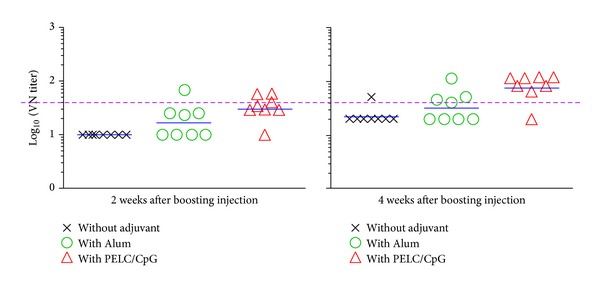
CVA16-specific VN antibody responses in vaccinated BALB/c mice. Three groups of mice (*N* = 8) were primed i.m. with 0.2 *μ*g of EV71 and 0.2 *μ*g of CVA16 combination vaccine, alone or formulated either with Alum or PELC/CpG. At week 14, mice were boosted i.m. with the same vaccine formulations. Serum samples were collected from vaccinated mice and the antibody titers were determined by VN immunoassay. The dotted horizontal line represents a VN titer of 40.

When the vaccinated mice were boosted with the same vaccine formulations at week 12, the CVA16-specific neutralizing antibodies at 4 weeks after boosting were found to be significantly increased in the PELC/CpG formulation group as shown in Figure 5 (*P* < 0.01). After the boosting dose, the VN titers were still undetected in most mice vaccinated with bivalent vaccine alone (Figure 5). In the Alum adjuvant group, the GMT of VN was found to be 20 and 40 at week 2 and week 4, respectively. PELC/CpG adjuvant bivalent vaccines were capable of inducing higher VN titers (GMT = 40  and 96 for weeks 2 and 4 after boosting, resp.) than those obtained from the Alum adjuvant group (*P* < 0.05). Thus the current results demonstrate that the antigen-specific antibodies can be significantly enhanced by a booster dose.

## 4. Discussion

EV71 and CVA16 are the two major enteroviruses causing HFMD and combine together contributing to over 50% of HFMD cases that recently happened in Asia [1–3]. Formalin-inactivated EV71 candidate vaccines currently being evaluated in clinical trials are most likely to be effective against different strain of EV71 since these vaccines have been shown to be protective in animal models and humans but failed to protect against CVA16 [4–9]. In addition, our study [9] and others [10] also showed that inactivated CVA16 candidate vaccines could elicit strong CVA16-specific neutralizing antibody responses but had no protection or virus neutralization against EV71. Taking these results together, a bivalent EV71/CVA16 vaccine should be developed to protect children from HFMD. It is well-known that formulation of multivalent vaccines is not easy due to immune interference that one immunogen in the combination vaccine is dominant over the others [20–22]. These imbalanced immune responses could be biased in protecting the immunodominant target virus [21, 22]. The imbalanced immune responses could be overcome by vaccine formulation, in particular formulated with a potent adjuvant [23]. A novel vaccine adjuvant is capable of inducing potent and broadened immune responses to overcome the chemical and immunological incompatibility interference in the vaccines combination [17, 23–26]. In this study, we therefore design and evaluate the immune responses of bivalent EV71/CVA16 candidate vaccine formulated with different adjuvant in mouse model.

In pandemic influenza vaccine preparedness studies, two squalene-based O/W emulsions, MF59 (Novartis) and AS03 (GlaxoSmithKline), have been successfully used to increase the efficacy, immunogenicity, and cross-protection of human vaccines [11]. Previously, we had reported the design, optimization, and application of a submicron multiphase (W/O/W) emulsion system, PELC, in vaccine development [13, 14]. Emulsified formulations have several advantages over traditional Alum formulation: they are stable, reproducible, and homogeneous fine submicron particles with an appropriate size to facilitate the induction of potent immune responses. At the beginning, we prepared emulsion compositions using different surfactants/excipient to optimize the W/O/W emulsification-dispersion procedure. The results (Figure 1) demonstrate that inactivated EV71 formulated with PELC-emulsified submicron particles induces more potent antigen-specific serum antibody responses than those obtained with a nonadjuvant EV71 vaccine. In addition, PELC-emulsified EV71 virion was found to be better than Alum formulation in eliciting mouse antibodies to recognize EV71 virus neutralization epitopes. The present study also shows that inactivated EV71 virion formulated with either PELC or PELC/CpG combination induces robust antibody immune responses compared with those obtained from inactivated EV71 virion alone. The combination of PELC/CpG adjuvant formulation nevertheless in this study has shown some advantage over PELC emulsion system that is capable of eliciting earlier neutralizing antibody responses (Figures 3 and 4). These results are consistent with our previous studies in which PELC/CpG combined a particulate delivery system (PELC) with an immunostimulatory compound (CpG) to provide sufficient danger/alarm signals to the immune cells [14, 17]. We also demonstrated again that the EV71 virion was antigenic distinguishable from the CVA16 virion and a single dose (0.2 *μ*g/0.2 *μ*g) of the bivalent EV71/CVA16 candidate vaccine is not sufficient to induce neutralizing antibody against CVA16. The induction of CVA16 neutralizing antibody responses requires a prime-boost vaccination schedule. Therefore, to protect against HFMD (EV71/CVA16 virus infections), a boost emulsified EV71/CVA16 bivalent vaccine is recommended.

Concerning the T-cell immunity, there is evidence that the poor EV71-specific cellular Th1 response may be associated with immunopathogenesis of EV71-related pulmonary edema [27]. Therefore, increasing cellular Th1 cytokine induction via vaccination is thought to be an important strategy for overcoming EV71 infections. The current results shown in Figure 4 have demonstrated that the bivalent EV71/CVA16 formulated with PLEC/CpG could induce Th1 bias immune response due to high ratio of IgG2a/IgG1 titer. We had attempted to determine whether T-cell responses could be manipulated in the inactivated EV71 virion formulated with designed adjuvant formulations. Our findings indicate that even at high dosage (1.0 *μ*g) of inactivated EV71 virion, the cytokine-secreting responses such as IFN-*γ*/IL-4-secreting cells (ELISPOT) and the IFN-*γ*/IL-4 concentrations in the splenocyte supernatants (ELISA) were found to be at the undetectable level regardless of the adjuvant formulations (data not shown). It still needs to be evaluated whether the formulation of formalin-inactivated EV71 virion with other potent adjuvant (toll-like receptor 2 and 4 agonists) is beneficial on the host immune cells. Further investigations are under way to examine the efficacy of the EV71/CVA16 bivalent vaccine formulation in protection against EV71 and CVA16 challenge using the human SCARB2-transgenic mice [28, 29], which have been shown to develop enterovirus-associated disease mimicking those seen in young children infected with the enterovirus.

## Figures and Tables

**Table 1:** The composition and particle size of the designed emulsion-based adjuvants.

Emulsion adjuvant	Components^a^	Particle size^b^ (Mean ± STD, nm)
PELC	Squalene, PEG-b-PLACL, Span 85	338 ± 129
PELC(T)	Squalene, Tween 80, Span 85	284 ± 17
PELC(A)	Squalene, Tween 80, *α*-tocopherol	407 ± 155

^a^Emulsification process was performed using homogenizer under 6,000 rpm for 5 min.

^b^Data were represented as the mean with standard deviation (STD) of three samples.
